# Military Working Dogs Operating in Afghanistan Theater: Comparison between Pre- and Post-Mission Blood Analyses to Monitor Physical Fitness and Training

**DOI:** 10.3390/ani12050617

**Published:** 2022-03-01

**Authors:** Giuseppe Spinella, Lorenzo Tidu, Lisa Grassato, Vincenzo Musella, Micheletino Matarazzo, Simona Valentini

**Affiliations:** 1 Department of Veterinary Medical Sciences, University of Bologna, 40064 Ozzano dell’Emilia, BO, Italy; giuseppe.spinella@unibo.it; 2 “Vittorio Veneto” Division Florence-NATO Multinational Division South, 50136 Firenze, FI, Italy; casezsanivet@dvttveneto.esercito.difesa.it; 3 Veterinary Clinic “Il Podere”, 31038 Postioma, TV, Italy; 4 Department of Health Sciences, University of Catanzaro, 88100 Germaneto, CZ, Italy; musella@unicz.it; 5 Italian Army Military Veterinary Center (CEMIVET), 58100 Grosseto, GR, Italy; caservet@cemivet.esercito.difesa.it

**Keywords:** military working dogs, explosive detention dog, humanitarian mission, working injury

## Abstract

**Simple Summary:**

In modern conflicts, one of the greatest risks for military personnel is represented by explosive devices. For this reason, specifically trained dogs able to detect explosives have been more and more intensively used in humanitarian military operations. However, at present, literature regarding working problems reported by these animals is very limited. The a of this study was to evaluate the health status of military dogs participating in humanitarian missions to Afghanistan, comparing their pre- and post-mission blood work. Dogs were first considered together as a group and then divided in groups by age, sex, breed and mission length. The results of the study show that there are no particular differences between their blood work before and after the missions. This indicates that the recorded dogs were physically well prepared to face their tasks.

**Abstract:**

The intergovernmental organization known as the United Nations (UN) was born “to maintain international peace and security” through different operations and tasks, including “mine action” and “explosive detection”. Explosives are the most frequent cause of injuries in military personnel and an enormous danger for civilians. The role of explosive detection dogs (EDDs) and mine detection dogs has gained great consideration over time, leading to their intense use in military operations. Literature regarding working injuries reported by EDDs during missions is limited. The aim of the present study is to investigate the hematological changes that occurred between pre- and post-mission blood analyses in military working dogs deployed to Afghanistan in order to evaluate signs of health problems or physical adjustments. Examining the clinical records, only three dogs reported a medical issue, one with gastric dilatation-volvulus (GDV), and two with lameness episodes. Lack of health issues occurring during the missions was reflected by the absence of significant differences between pre- and post-mission blood analyses. Blood results were also examined by dividing the EDDs into groups considering age at departure, sex, breed and mission length. A few categories demonstrated significant changes in some parameters; however, the mean values were always included in the ranges of normality, indicating that their physical fitness and training were adequate for the required tasks.

## 1. Introduction

Military working dogs (MWDs) have been involved during peacekeeping operations alongside of military soldiers, and they are trained for specific jobs, including tracking, explosion detection, patrol, search and rescue, and attack [1].

The tasks of the explosive detection dogs (EDDs) are increasingly specialized and refined, and their breeding, training, maintenance and preparation require extensive time and economic investment [2]. A general EDD is trained to search at the proximal direction of a handler, generally on lead. The area of search can involve several settings, such as buildings, restricted open areas, vehicles and boxed goods. Expansions of these common duties represent more sophisticated applications: searches more focused on a particular task or in a particular context, or variations in the method by which the search is executed. For example, specialized search dogs can be remotely-directed, they can work on specific main route clearance, land-mine, or cargo screening. Some search duties can also be defined by the required amount of explosive scent to be identified: in the aviation security sector, it is vital for EDDs to detect trace levels of explosives, while in a combat theatre EDDs may need to be trained to ignore trace amounts of explosives [2]. MWDs, and particularly detection dogs, need multiple physical and behavioral qualities: great scent sense and innate social-cognitive skills are essential, along with an appropriate physical and fitness training and education to maintain motivation and engagement to the task [2,3,4,5,6]. Altitude hypoxia and high-temperature environments, for example, may divert the dog’s attention from the searching task, also because dogs are physically unable to sniff and pant simultaneously, decreasing the olfaction and detection efficiency: detector dogs should be athletic and well trained to be able to cope with challenging search environments [3,4,6]. High-altitude adaptation also includes increased oxygen uptake and delivery, determined by higher hemoglobin (HGB) concentration and oxygen affinity, or by high level of blood flow [7,8]. MWDs should be prepared for exposure to high temperatures, and handlers should be trained to recognise and effectively handle heath stress. Familiarisation of the MWDs with environments similar to the area of operation would assist their adjustment to it. 

Another important contemplation is the evaluation of the operation requirements (rest and work schedules, number of dogs needed to effectively fulfil the mission) [9,10]. The health and welfare of the MWDs are crucial, and they are kept in excellent fitness with regular courses and specific training. Generally, MWDs work 8–12 h a day, many days a week and they are fed a regular diet [11]. Pre-deployment examinations are vital to check the fitness-for-duty status of the MWDs [10]. The breeds most traditionally utilised as MWDs are German Shepherds and Belgian Malinois: intelligence, the adaptability to different situations, good detection capabilities and a successful rate at training for several military tasks are the main qualities attributable to these breeds [10,12,13,14,15,16,17,18]. 

Literature regarding working injuries reported by MWDs during missions is limited [12]. Together with gunshot, explosive and muscle-skeletal injuries [15,16], frequent medical problems are represented by trauma, heat stress and gastric dilatation and volvulus [10]. A further hazard reported for search-and-rescue dogs working in natural or man-made catastrophic events is the exposition to toxic agents such as smoke and products of combustion [17]. Furthermore, vector-borne diseases are a concern for MWDs, so intense preventative vector-control measures should be deployed [10]. This is particularly important also for public health as some vector-borne diseases represent possible zoonoses, such as Erlickiosis, Ricketiosis, Lyme disease and Leishmaniasis.

The aim of the present study is to investigate the hematological changes that occurred between pre- and post-mission blood analyses in EDDs deployed to Afghanistan, to evaluate signs of potential health problems or physical adjustments reported during the mission, and to indicate which variables may intervene more on them. To the authors’ knowledge, this is the first study regarding these settings. 

## 2. Materials and Methods

A standing agreement between the Italian Army (Esercito Italiano) and the Department of Veterinary Medicine of Bologna University for the use of the data below and for the development of the present study has been stipulated (f.n. M_D SSMD REG2020 0051733—27/03/2020 SMD –IGESAN). The study was also part of a wide project approved by the Ethical Committee of the University of Bologna on working and sports dogs (protocol number: ID 914/2018).

The Military Veterinary Center (CEMIVET) provided the clinical records of all EDDs participating in a humanitarian mission in the Middle-East in the 10-year-period 2010–2019. Inclusion criterium was geographical in order to reduce variables due to different environmental settings: all dogs participating in a mission in Afghanistan were considered. All EDDs experienced a similar life: before the mission and during the working hours, they were trained at the CEMIVET following a daily exercise routine consisting in open air walks and specific training, led by the respective handlers, interspersed by moments at rest, individually accommodated in large indoor or outdoor cages. Most EDDs returned home with their handlers at the end of the working day. All the EDDs attended learning and maintenance training based on olfactory research activities, often including updated courses for the research of new explosives. Dogs were fed with premium or super-premium categories of commercial food.

The outine activity of these dogs during the mission was as a barrier at the entrance of NATO military bases for cargo investigation; explosives detection activities outside the bases were also occasionally performed.

All EDDs were physically checked by a licensed military veterinarian, and medical fitness for the missions was attested before each operation. A blood sample was obtained from all dogs at the clinical examinations performed before the mission and at their return home, generally within 24 h from departure. The parameters tested included white blood cells, red blood cells, hemoglobin, hematocrit, platelets, mean cell volume, mean cell hemoglobin, mean corpuscular hemoglobin concentration, red cell distribution width, mean platelet volume, lymphocytes, monocytes, granulocytes, glucose, blood urea nitrogen, creatinine, aspartate transaminase, alanine aminotransferase, total proteins, albumine, total cholesterol, amylase, gamma glutamyl transeferase, creatine kinase, total bilirubin, lactate dehydrogenase and alkaline phosphatase, as specified in Table 1.

From a Public Health point of view, all EDDs were also tested for vector-borne diseases (Erlickiosis, Ricketiosis, Leishmaniasis), both pre- and post-mission, to monitor the possible role of the dogs as exporters of these pathologies.

Data regarding the presence or absence of any orthopaedic, gastroenteric or different conditions presented at any point during the working life of the EDDs were also acquired.

All data were submitted to descriptive (mean ± standard deviation-SD) and analytic statistical analysis. Analytic analyses to evaluate differences between the pre- and post-mission blood results were carried out for the whole group of EDDs. Subsequently, differences were categorized for age at the deployment (younger and older than the mean age), sex (males and females), breed (German Shepherds and Belgian Malinois) and mission length (shorter and longer than the calculated mean duration). All data were previously submitted to a Shapiro-Wilk test for evaluation of normality distribution. The unpaired *t*-test or Mann-Whitney U test were used for statistical analyses if no normal distribution was reported in order to investigate any significant variation between before and after the mission. The significance for all tests was set at *p* < 0.05.

All of the statistical analysis was performed using R Core team (2020) software for Mac.

## 3. Results

Thirty-five EDDs were included in the study: 17 of them participated in more than one mission in Afghanistan, for a total of 54 registered cases. The breed distribution comprised four Belgian Malinois (seven missions) and 31 German Shepherd Dogs (47 missions); 25 were males and 10 females. Mean age at the beginning of the missions was 52.60 months (SD 20.37; range 22–108 months).

The missions were distributed in several NATO bases in Afghanistan and the mean duration was 6.43 months (SD 1.66; range of two to nine months).

The mean and SD of the included EDDs for all the considered parameters, both pre- and post-mission, are reported in Table 1.

When considering the whole group of included EDDs, no significant differences were found for any of the measured parameters between the values recorded pre- and post-mission.

On the other hand, in categorizing the EDDs by breed, sex, age at the deployment and mission length, some statistically significant differences were recorded for the following groups and analytes: red blood cells distribution width for Malinois breed, lymphocytes for males, HGB for younger dogs, glucose for elders, monocytes for shorter missions, and lymphocytes and granulocytes for longer deployments.

Despite the significant differences, all the parameters remained in the respective range of normality for all the indicated categories.

None of the EDDs presented clinical disorders before or after their mission deployment requiring an external laboratory to process their blood work, so the general blood work was conducted in the military center’s internal laboratory: due to the retrospective nature of the study and the different availability of substrate reagents for the several analytes from time to time, some parameters were reported only for a low number of cases (i.e., total cholesterol tCHOL, amylase AMY, gamma glutamyl transeferase GGT, creatine kinase CK, total bilirubin tBIL, lactate dehydrogenase LDH, alkaline phosphatase ALP) and the statistical analyses deriving from them were carefully considered as indicated in the discussion section.

Two dogs tested positive for Erlichiosis (sero-prevalence of 1/80 and 1/160, respectively) pre-mission; the first resulted negative at its return home and the second was positive with a sero-prevalence of 1/80. One dog tested positive for leishmaniasis post-mission (sero-prevalence 1/320).

One episode of gastric dilatation and volvulus (GDV) was registered during the missions and referred to a male German Shepherd dog, older than the mean age at the beginning of the mission, which participated in the mission for a duration shorter than the mean mission length.

Two dogs demonstrated acute lameness of the left hindlimb. Both were male German Shepherd dogs, younger than the mean age, participating in their mission for a period longer than the mean mission durations.

## 4. Discussion

To the authors’ knowledge, this is the first study aiming at the examination of potential variations in the blood analyses of EDDs deployed to a mission and investigating possible signs of health issues or physical adjustments to the environment and life. The tasks of these EDDs included routine investigations of all cargo and vehicles entering their NATO military bases and occasional explosives detection activities outside of the bases.

Based on a literature review, only limited data are available on working injuries reported by MWDs during missions, and the most frequent problems reported are gunshot or explosive injuries, muscle-skeletal damage, heat stress and GDV [10,12,15,16]. Examining the clinical records of the EDDs, only three dogs reported a medical issue that occurred during the missions: one GDV and two lameness episodes.

It is reported in the literature that the habituation of the MWDs to environments similar to the area of mission can contribute to their adjustment to it [10]. The geographical area of deployment has a continental climate, with temperatures to which dogs are generally used to: this can help in preventing heat stress, together with the education of the handlers on recognition of the risk factors and on measures to prevent it [10]. Furthermore, the usual activity executed by the dogs (vehicles investigation) required a great work for short periods of time, which could have helped them against heat stress and muscle-skeletal chronic damages due to cumulative micro-traumas from overuse [12]. May et al. [12] reported that the oldest MWDs included (age ≥ 80 months) registered increased odds of musculo-skeletal injuries; the mean age of the EDDs of the present study was 52.60 months, so it is possible that younger dogs are at lesser risk for these specific injuries. However, in the present study, controversially, the only two dogs that reported lameness during the mission were dogs younger than the mean population age. The possible lameness cause was overuse, but it was not better speculated. In both cases it resolved with the administration of NSAIDs. It is possible that younger dogs may be less experienced and more active than older dogs, and therefore more exposed to traumatic injuries [18]; this is in contrast with the indications of the previously cited paper, and further investigations with wider populations would be recommended.

Risk factors for GDV are controversial; however, stress, alimentary and exercise habits may contribute [19], potentially suggesting that the physical and mental status and regimens of these dogs were adequate, as only one dog presented this problem during the mission.

A further concern for MWDs is represented by vector-borne diseases [10]. This becomes particularly important as a public health issue, as some of these diseases could represent zoonoses, and it is vital to ensure the safety of the dogs and people involved in the missions, as well as to avoid their possible role as exporters of these pathologies. The EDDs of this study underwent strict and continuous vector control regimens, including monthly oral or topical administration of heartworm and intestinal parasite anthelmintic preventive treatment. Prophylactic measures against external parasites (such as ticks, fleas and sandflies) were continuously provided through apposite collars or the monthly application of topic spot-on agents. Among 54 analyzed cases, no EDDs contracted Ricketiosis or Erlichiosis during the missions, possibly indicating that preventative vector-control measures proved effective. However, two dogs tested positive for Erlichiosis pre-mission and one dog tested positive for leishmaniasis post-mission. This might indicate that wider health protocols or combinations of the existent ones should be considered; it might also be worth considering a more efficient training of the handlers on how to effectively apply the prophylactic measures. In this regard, a prospective and wider study should be performed to better frame these important problems.

Lack of health issues occurring during the missions was reflected by the absence of significant differences between pre- and post-mission blood analyses values in the cohort of EDDs included in the study. The only parameter showing a change was the LDH, which decreased after the mission; however, this parameter was registered only for a very low number of cases (10 pre- and 17 post-mission) and subsequently its variation was not deemed valid to produce strong and reliable observations.

The elevation of plasmatic muscle enzymes such as CK, AST, and LDH can suggest muscular injury and fatigue [20,21,22,23,24,25,26,27,28]. High plasmatic concentrations of these enzymes are due to the increased permeability and damages of the skeletal muscle cellular membranes, and they can be used to diagnose muscular pathological conditions. In healthy dogs their elevation can be temporary and may indicate a physiological response to intense exercise [23,27,28,29,30,31]. Two studies on search and rescue dogs [20,32], for example, reported increases in CK, AST and LDH due to exercise, but these parameters decreased to baseline after a rest of 30 min to 2 h. Different variables may affect the entity of the plasmatic elevations of these parameters, such as age, sex, breed and training [28,29,33].

In the present study, blood results were also examined by dividing the EDDs in groups considering age at the departure for the mission, sex, breed and mission length: once again, regardless of the group, no significant differences were spotted in these muscle enzymes’ plasmatic concentrations between their pre- and post-mission values. It is worth considering that the variability of the blood analytes related to their measurement time might represent a limit to these results.

Changes in serum levels of glucose and non-esterified fatty acids can also suggest glycogen storage capacity in muscle [20,21]. In this study, non-esterified fatty acid was not recorded, however, EDDs of older age demonstrated an elevation of glucose concentration post-mission; this may suggest that their skeletal muscle energy metabolism is less efficient than in the younger ones; however, once again, the number of data recorded in this regard are quite limited (16 pre-mission, 20 post-mission) and this observation would require further investigations, particularly because this category of dogs did not demonstrate any other signs of muscle fatigue or damage.

The absence of variations in plasmatic levels of these parameters may indicate that the dogs were not subjected to strenuous exercise and they did not suffer significant fibrillary disruption and long-term damages; this also implies that their physical fitness and training were adequate for the required tasks.

EDDs included in this study were all German Shepherd (31 dogs; 47 missions) or Belgian Malinois (four dogs; seven missions) dogs, in accordance with MWDs breed prevalence reported in the literature [10,12,13,14,15]. A comparison between pre- and post-mission blood results was executed for the two breeds separately: no significant differences were reported by German Shepherd dogs for any of the registered parameters, whereas a significant decrease of RDW concentration has been registered for Belgian Malinois dogs. It is the authors’ belief that this decrease is not of any relevance, as it was not correlated to any other hematological or clinical abnormalities and the registered values were within the normality range anyway; furthermore, only four dogs represented this breed and the very low numbers registered may create a limit to the reliability of the data. The breed prevalence and lifestyle of these dogs might represent risk factors for muscular diseases such as gracilis muscle contracture, a pathology most often encountered in active, adult German Shepherd dogs [34]; however, this problem was not registered by any of the EDDs included in the study.

High-altitude adaptation can induce some physiological changes including increased HGB concentration and oxygen affinity or high levels of blood flow [7,8]. In the present study, a significant increase of post-mission HGB concentration was only registered by the younger EDDs, although variation values were within the physiological range of normality. The altitude of the NATO bases where these dogs were deployed ranged between 500 and 1500 m above sea level. In the human literature, it is indicated that some physiological adaptations can be seen from relatively low altitudes such as 1000 m above sea level, and they become more evident from 2000 m above sea level [35]. If altitude adaptation was the cause of the HGB elevation in the post-mission values for the younger EDDs, it is not clear why this was not found for older EDDs as well. However, a physiological increase of HGB can also be seen in working and sportive dogs, and this is secondary to exercise due to the release of splenic and medullar reserves [36].

EDDs involved in a mission for longer than the calculated mean duration of all the missions demonstrated a significant increase of lymphocytes and granulocytes in the post-mission values. These findings might relate to the trend of a possible stress leukogram and might suggest that dogs deployed for longer periods may start experiencing stress more than EDDs deployed for a shorter period. However, once again, the mean values registered were within the reference ranges for dogs, possibly indicating that the dogs were coping well with it. However, EDDs deployed for shorter missions registered a significant increase of monocytes, which might still be related to stress, possibly because they faced two voyages (transfer to and from the mission base) in a short period of time, and the second leg of the travel might have caused a higher level of stress as emotive anticipation.

Male dogs recorded a significant decrease of lymphocytes post mission. These data are difficult to explain; however, it should be kept in mind that all of these changes, although statistically significant, were inside the physiological ranges.

This study presents some limitations, mainly deriving from its retrospective nature, which did not allow for the registering of all the reported parameters for all the EDDs or to add some different variables to the blood analyses that might have been helpful to better define some conditions, such as lactates and fatty acids, for example.

## 5. Conclusions

It is quite clear from the results of the present study that the group of EDDs included did not show any critical clinical or hematological signs of health problems suffered during their deployment on the missions, reflecting an adequate and effective management and physical training. The authors believe that this paper may represent a first, preliminary contribution to the scientific community in the study and improvement of the consideration of health and wellbeing in MWDs, particularly with regard to the importance of their training and behavioral management. Furthermore, prospective studies would be suggested to increase the reliability of our findings in different categories of working dogs.

## Figures and Tables

**Table 1:** Parameters tested and the number of cases they were available for. (WBC white blood cells, RBC red blood cells, HGB hemoglobin, HCT hematocrit, PLT platelets, MCV mean cell volume, MCH mean cell hemoglobin, MCHC mean corpuscular hemoglobin concentration, RDW red cell distribution width, MPV mean platelet volume, LYM lymphocytes, MON monocytes, GRA granulocytes, GLU glucose, BUN blood urea nitrogen, CREA creatinine, AST aspartate transaminase, ALT alanine aminotransferase, TP total proteins, ALB albumine, tCHOL total cholesterol, AMY amylase, GGT gamma glutamyl transeferase, CK creatine kinase, tBIL total bilirubin, LDH lactate dehydrogenase, ALP alkaline phosphatase). SD: standard deviation. No significant statistical differences between pre- and post-mission were observed.

Parameter	Pre-Mission			Post-Mission		
	Registered Cases	Mean	SD	Registered Cases	Mean	SD
WBC (10^3^/mm^3^)	44	7.9173	1.8421	49	7.5569	2.2657
RBC (10^3^/mm^3^)	44	7.5873	0.8982	51	7.4582	0.8296
HGB (%)	44	16.8973	2.3405	52	17.4023	2.2678
HCT (%)	44	51.1945	6.5800	52	51.1256	5.6880
PLT (10^3^/mm^3^)	42	273.6429	114.8256	50	253.6200	74.8876
MCV (fL)	42	82.7588	93.8252	47	66.4785	9.4234
MCH (pg)	42	23.1188	2.0549	47	24.5523	8.9462
MCHC (%)	42	33.9307	2.9219	47	34.2740	2.3454
RDW (%)	41	14.8405	1.4370	46	15.4028	2.5762
MPV (fL)	40	9.3310	1.8348	44	9.0211	1.6699
LYM (10^3^/mm^3^)	43	1.6630	0.6576	50	1.5382	0.6822
MON (10^3^/mm^3^)	43	0.3712	0.2163	51	0.4859	0.5266
GRA (10^3^/mm^3^)	41	6.0290	1.4038	50	5.3928	1.7960
GLU (mg/dL)	16	84.6875	15.3089	20	91.750	9.0895
BUN (mg/dL)	23	26.96	15.426	32	24.31	13.881
CREA (mg/dL)	22	1.155	0.1862	30	1.1217	0.2606
AST (U/L)	19	26.79	13.624	29	31.31	15.421
ALT (U/L)	17	62.823	45.2372	32	58.125	42.6552
TP (g/dL)	21	6.3238	0.5804	32	6.4406	0.5874
ALB (g/dL)	19	2.6321	0.5537	32	2.8450	0.4889
tCHOL (mg/dL)	15	274.13	66.666	20	268.45	72.610
AMY (U/L)	9	785.67	74.024	9	714.44	229.145
GGT (U/L)	11	12.73	7.115	8	7.13	5.111
CK (U/L)	7	209.43	145.305	11	124.82	68.353
tBIL (mg/dL)	12	0.3942	0.1889	13	0.2785	0.0950
LDH (U/L)	10	220.40	215.883	17	102.71	56.167
ALP (U/L)	10	43.30	18.031	17	52.41	27.543

## Data Availability

All data are contained in the manuscript.
